# Intrathecal levels of matrix metalloproteinases in systemic lupus erythematosus with central nervous system engagement

**DOI:** 10.1186/ar1228

**Published:** 2004-09-23

**Authors:** Estelle Trysberg, Kaj Blennow, Olof Zachrisson, Andrej Tarkowski

**Affiliations:** 1 Department of Rheumatology and Inflammation Research, Göteborg University, Sahlgrenska University Hospital, Göteborg, Sweden; 2 Institute of Clinical Neuroscience, Göteborg University, Sahlgrenska University Hospital, Göteborg, Sweden

**Keywords:** cerebrospinal fluid, magnetic resonance imaging of the brain, matrix metalloproteinase-2, matrix metalloproteinase-9, neuropsychiatric involvement in systemic lupus erythematosus

## Abstract

Symptoms originating from the central nervous system (CNS) occur frequently in patients with systemic lupus erythematosus (SLE), and CNS involvement in lupus is associated with increased morbidity and mortality. We recently showed that neurones and astrocytes are continuously damaged during the course of CNS lupus. The matrix metalloproteinases (MMPs) are a group of tissue degrading enzymes that may be involved in this ongoing brain destruction. The aim of this study was to examine endogenous levels of free, enzymatically active MMP-2 and MMP-9 in cerebrospinal fluid from patients with SLE. A total of 123 patients with SLE were evaluated clinically, with magnetic resonance imaging of brain and cerebrospinal fluid (CSF) analyses. Levels of free MMP-2 and MMP-9 were determined in CSF using an enzymatic activity assay. CSF samples from another 22 cerebrally healthy individuals were used as a control. Intrathecal MMP-9 levels were significantly increased in patients with neuropsychiatric SLE as compared with SLE patients without CNS involvement (*P *< 0.05) and healthy control individuals (*P *= 0.0012). Interestingly, significant correlations between MMP-9 and intrathecal levels of neuronal and glial degradation products were noted, indicating ongoing intrathecal degeneration in the brains of lupus patients expressing MMP-9. In addition, intrathecal levels of IL-6 and IL-8 – two cytokines that are known to upregulate MMP-9 – both exhibited significant correlation with MMP-9 levels in CSF (*P *< 0.0001), suggesting a potential MMP-9 activation pathway. Our findings suggest that proinflammatory cytokine induced MMP-9 production leads to brain damage in patients with CNS lupus.

## Introduction

Central nervous system (CNS) involvement has been reported to occur in 14–75% of all systemic lupus erythematosus (SLE) patients [1-3]. However, frequency rates vary considerably, depending on the diagnostic criteria applied. CNS lupus can occur at any time during the course of SLE, and its symptoms are diverse. The features of this condition can include seizures, stroke, depression, psychoses and disordered mentation. Neuropsychiatric involvement in SLE (NPSLE) has been shown to predict a high frequency of flares, and it is considered a major cause of long-standing functional impairment as well as a cause of mortality [4]. Over the past decade CNS lupus has been treated with cytotoxic drugs, which improve disease outcome [5,6].

Because of the multiple pathogenic mechanisms that cause manifestations of CNS lupus, no single confirmatory diagnostic test is available. Several clinical, laboratory and radiographic test findings are reported to be abnormal in some but not all patients with CNS lupus. Magnetic resonance imaging (MRI) of brain has been shown to be valuable in detecting even minor lesions caused by CNS lupus, and these correlate with CNS manifestations in SLE [7]. Pleocytosis and elevated protein levels are found in some but not all patients with CNS lupus [8,9]. Elevated concentrations of IgG in cerebrospinal fluid (CSF), IgG:albumin ratio and IgG index, and the presence of oligoclonal bands have all been described with varying frequencies in patients with NPSLE [10-12]. Few studies have demonstrated elevated IL-6 levels in CSF from patients with CNS lupus [13-17]. Some other reports have described increased levels of IL-1 [13], IL-8 [15] and interferon-γ [18] in CSF from patients with CNS lupus. We recently reported neuronal damage, astrogliosis and formation of toxic metabolic products such as Aβ42 in patients with NPSLE [19].

One of the prototypical destructive events in the human brain, initiated by the release of inflammatory cytokines and ending with tissue destruction, is production of matrix metalloproteinases (MMPs). The MMPs are a family of endopeptidases produced by a variety of inflammatory cells [20]. All of the cell types that exist in the CNS are potential sources of MMPs. *In vitro*, neurones, astrocytes, microglia [21,22] and oligodendrocytes [23] express various MMP family members, and production of MMPs by neuronal cells can be upregulated by several inflammatory cytokines. MMPs have a multitude of regulatory functions, including control of influx of inflammatory mononuclear cells into the CNS, participation in myelin destruction and disruption of the integrity of the blood–brain barrier [24]. With respect to MMP-9, it was recently shown that CSF levels of this enzyme increase during bacterial meningitis and that it is associated with brain damage [25,26]. The aim of the present study was to measure levels of free active MMP-2 and MMP-9 in CSF of SLE patients with CNS lupus, and to relate these data to clinical and laboratory measures of brain parenchymal degradation. Our results suggest that MMP-9 but not MMP-2 actively participates in brain destruction in CNS lupus.

## Methods

### Participants

A total of 122 patients fulfilled four or more of the American Rheumatism Association 1987 revised criteria for the classification of SLE [27]. The patients (106 females and 16 males, aged 17–91 years [mean age ± standard deviation 42 ± 14 years]) were being treated at the Department of Rheumatology at Sahlgrenska University Hospital. Disease duration varied between 0 and 49 years (mean duration 8 ± 9 years). The patients were consecutively included in the study. They underwent a thorough clinical examination by experienced staff rheumatologist, neurologist and neuropsychologist. Examination of CNS signs and symptoms included lumbar puncture, neuropsychological tests and MRI of the brain.

The proposed definition of CNS lupus in the American Rheumatism Association criteria for SLE [27] appears inadequate, given that only two elements – psychosis and seizures – are included. As previously described [28], we defined CNS lupus as the presence of at least two of the following seven items in association with clinical evidence of disease progression: recent onset psychosis, transverse myelitis, aseptic meningitis, seizures, pathological brain MRI, severely abnormal neuropsychiatric test findings [29] and oligoclonal IgG bands in CSF. The pathogenesis of antiphospholipid antibody mediated brain damage is a thrombotic rather than inflammatory complication of SLE, and so we decided to exclude this condition from the definition of CNS lupus. Non-SLE causes of neurological events (e.g. cerebral infections) were also ruled out. Based on the criteria above, patients were divided into three distinct groups: patients with CNS lupus (*n *= 43); patients with SLE but without any signs of CNS involvement (*n *= 71); and patients with SLE complicated by antiphospholipid syndrome or patients who met the CNS lupus criteria but were ruled out because of non-SLE origin of neurological events (*n *= 9). The frequencies of various CNS manifestations and patient treatments in the SLE patients are summarized in Tables 1 and 2, respectively. The study was approved by the ethical committee of the University of Göteborg.

### Control individuals

CSF from 22 healthy individuals (mean age ± standard deviation: 38 ± 11 years; 12 females and 10 males), without previous history of neurological disorder and with normal neurological status, served as control individuals. There were no significant differences between males and females with respect to intrathecal levels of MMP-9 (0.17 ± 0.17 pg/ml versus 0.30 ± 0.30 pg/ml; not significant) or intrathecal levels of MMP-2 (388 ± 48 pg/ml versus 379 ± 48 pg/ml; not significant).

### Cerebrospinal fluid analyses

Levels of MMP-2 and MMP-9 were determined using an activity assay system (Amersham Pharmacia Biotech, Buckinghamshire, UK), which was constructed to measure enzymatically active forms of MMP-2 and MMP-9. The detection level was 190 pg/ml for MMP-2 and 125 pg/ml for MMP-9. All values below the detection levels were considered negative. Paired serum and CSF samples were analyzed for albumin and IgG levels using nephelometry. As an indicator of blood–brain barrier function, the quotient of CSF albumin × 10^3^/serum albumin was analyzed (normal values <6.8 [<45 years of age] and <10.2 [>45 years of age]) [30]. The CSF/serum IgG index was used as a measure of intrathecal IgG production and calculated by using the following formula (normal value <0.7): (CSF IgG × 10^3^)/([CSF albumin × 10^3^]/serum albumin). All CSF samples were also analyzed by isoelectric focusing to permit detection of oligoclonal IgG bands.

### Magnetic resonance imaging analyses

Neuroimaging was performed to evaluate the extent and localization of brain lesions. The neuroimaging technique used was multiplanar MRI. The MRI examinations (Philips Gyroscan T5-II, Einhoven, The Netherlands) were performed with axial proton density and T_2_-weighted images of the brain. MRI abnormalities were seen in 72% of patients with CNS lupus and in 33% of SLE cases classified as cerebrally healthy.

### Statistical analysis

Statistical comparisons were made using the nonparametric Mann–Whitney U-test or, in case of follow-up data, the Wilcoxon's test for paired data. Results are presented as means ± standard error of the mean. *P *< 0.05 was considered statistically significant. Spearman-rank correlation was used for calculation of correlation. The statistical analyses were conducted using the Statview^® ^(SAS, Cary, NC, USA) program.

## Results

A total of 123 patients met criteria for SLE. Forty-three patients were found to have NPSLE (in accordance with the criteria presented in the Methods section above), and nine patients either were found to have phospholipid antibody syndrome or met the CNS lupus criteria but were excluded because of non-SLE origin of neurological events. The remaining 71 SLE patients were considered to be cerebrally healthy.

Mild pleocytosis was seen in patients with CNS lupus (9 × 10^6 ^± 6 × 10^6 ^cells/l) as compared with cerebrally healthy SLE patients (2 × 10^6 ^± 0.5 × 10^6 ^cells/l; not significant). As previously validated [31], we found an increased number of oligoclonal bands in CSF from the CNS lupus group (1.7 ± 0.4) as compared with SLE patients without CNS involvement (0.5 ± 0.1; *P *< 0.05). The mean level of CSF:serum albumin ratio was not increased in patients with NPSLE (6.1 ± 0.7 mg/dl) as compared with cerebrally healthy SLE patients (5.2 ± 0.3 mg/dl; not significant). There were no significant differences in levels of serum antibodies specific for native DNA or in complement levels (C3 and C4) between SLE patients with and those without CNS involvement.

**Figure 1:**
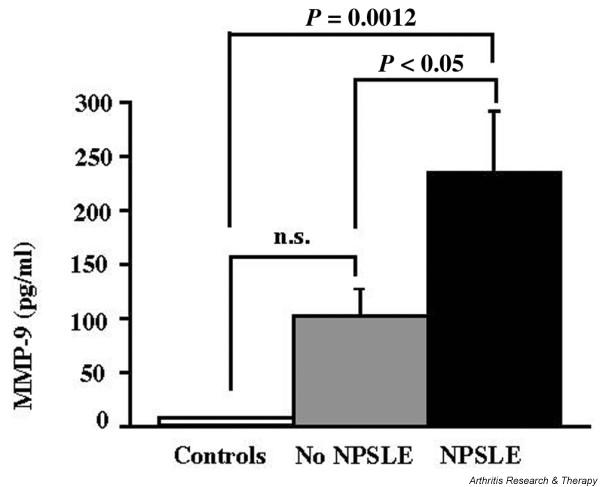
Cerebrospinal fluid expression of active matrix metalloproteinase (MMP)-9 in patients with systemic lupus erythematosus with (NPSLE) and without (No NPSLE) central nervous system involvement, and in cerebrally healthy control individuals.

Intrathecal MMP-9 levels were significantly increased in all SLE patients as compared with cerebrally healthy control individuals (153 ± 27 versus 0.28 ± 0.16 pg/ml; *P *= 0.016). In CNS lupus patients MMP-9 levels were significantly increased, both compared with cerebrally healthy SLE patients (240 ± 60 versus 100 ± 20 pg/ml; *P *< 0.05; Fig. 1) and compared with cerebrally healthy control individuals (240 ± 60 versus 0.28 ± 0.16 pg/ml; *P *= 0.0012). On stratification of all SLE patients into two groups with respect to the presence or absence of brain MRI pathology, we found increased CSF MMP-9 levels in SLE patients with MRI pathology as compared with those without (160 ± 50 versus 140 ± 30 pg/ml; not significant).

**Figure 2:**
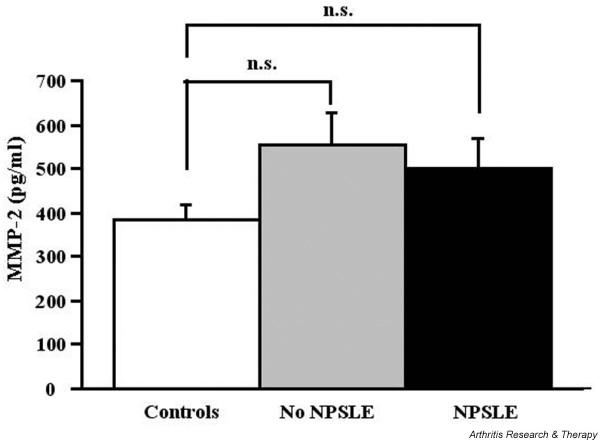
Cerebrospinal fluid expression of active matrix metalloproteinase (MMP)-2 in patients with systemic lupus erythematosus with (NPSLE) and without (No NPSLE) central nervous system involvement, and in cerebrally healthy control individuals.

There were no statistically significant differences in CSF MMP-2 levels between NPSLE and patients without CNS lupus (499 ± 70 versus 555 ± 70 pg/ml; not significant) or compared with cerebrally healthy control individuals (499 ± 70 versus 384 ± 33 pg/ml; not significant; Fig. 2).

**Figure 3:**
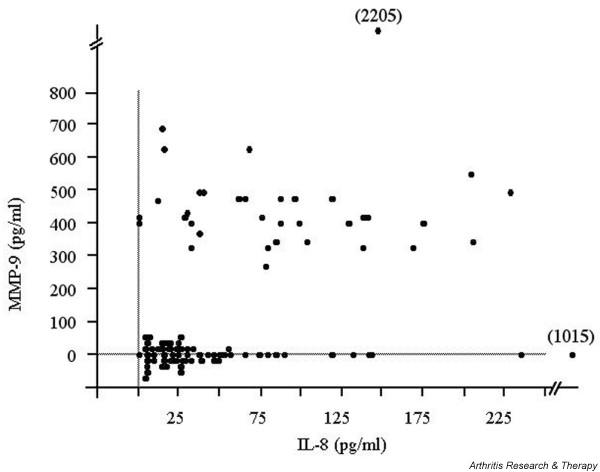
Scattergram showing relationship between IL-8 and matrix metalloproteinase (MMP)-9 in cerebrospinal fluid of all patients with systemic lupus erythematosus (r = 0.47; *P *< 0.0001).

CSF levels of IL-6 (47 ± 25 pg/ml versus 15 ± 3 pg/ml; *P *< 0.008) and IL-8 (91 ± 23 pg/ml versus 45 ± 6 pg/ml; *P *< 0.05) were both significantly increased in CSF from patients with CNS lupus as compared with cerebrally healthy SLE patients, supporting our previous findings [15,19]. Importantly, intrathecal levels of IL-6 and IL-8 significantly correlated with those of MMP-9 (r = 0.30 [*P *< 0.002] and r = 0.47 [*P *< 0.0001], respectively; Table 3 and Fig. 3). A neuronal degeneration marker (protein tau) and an astrocytic degeneration marker (glial fibrillary acidic protein) were both significantly increased in CSF from NPSLE patients as compared with CSF from SLE patients who were clinically free from CNS involvement (311 ± 78 pg/ml versus 178 ± 16 pg/ml [*P *< 0.05] and 1288 ± 708 pg/ml versus 396 ± 30 pg/ml [*P *< 0.009]), in concordance with previous findings [28,32]. Importantly, a significant correlation was noted between intrathecal MMP-9 and levels of tau and glial fibrillary acidic protein (*P *< 0.05 in both cases; Table 3).

## Discussion

We previously reported that patients with CNS lupus express increased intrathecal levels of proinflammatory cytokines IL-6, IL-8 and interferon-γ. Furthermore, we recently demonstrated that inflammation in CNS during SLE leads to neurodegeneration, which manifests as increased levels of neuronal and astrocytic degradation products [28,32]. However, the mechanisms responsible for the brain damage occurring during CNS lupus have never been clarified. It was previously demonstrated [33,34] that proinflammatory cytokines are able to trigger production and release of tissue-derived MMPs. It is also established that most of the resident cells in human brain have the capacity to produce MMP-2 and MMP-9 – enzymes that are known to participate in brain damage (e.g. in case of infectious meningitis) [35-37].

In the present study we found significantly higher levels of MMP-9 in CSF from NPSLE patients than in CSF from SLE patients without CNS lupus and healthy control individuals. This finding may be of clinical significance because of correlation between MMP-9 and levels of neuronal/astrocytic degradation products in CSF, reflecting the potential of MMP-9 to damage brain parenchyma. Findings of a relationship between MMP-9 and brain damage in SLE are new and should be scrutinized critically. We believe that effort should be invested in analyzing free (i.e. non-tissue inhibitor of metalloproteinase bound) and metabolically active enzyme to ascertain the validity of our conclusions. The method used in the present study fulfils both of these requirements.

MMP-9 plays an essential role in the breakdown of extracellular matrix molecules at the blood–brain barrier. This, together with locally elevated levels of the chemokine IL-8, greatly facilitates the transmigration of activated inflammatory cells across the endothelium. Indeed, a significant correlation was observed between the intrathecal levels of MMP-9 and IL-8.

A broad range of cytokines that are expressed in viral meningitis are able to regulate the production of MMPs and tissue inhibitors of metalloproteinase [38,39]. IL-1α and IL-1β, as well as IL-6, induce or upregulate the transcription of MMP genes [40]. This, along with increased IL-6 levels in CSF of patients with CNS lupus, may provide an explanation for the local synthesis of MMP-9.

CSF levels of enzymatically active MMP-2 were not increased in NPSLE patients as compared with SLE patients without CNS involvement or compared with cerebrally healthy control individuals. This finding is consistent with a previous study of viral meningitis, in which increased expression of MMP-9 but constitutive expression of MMP-2 was found [37]. The reason for this discrepancy, seen in the present study as well as in the previous one, might be differential regulation of gene promotors for MMP-9 and MMP-2. Indeed, whereas the promotor regions of MMP-9, which is regulated by cytokines, harbor a nuclear factor-κB responsive element, those of MMP-2 lack a TATA box and contain two SP-1 sites, both characteristic of house keeping genes [40,41].

## Conclusion

Our present and previous findings indicate that the following chain of events takes place during NPSLE: intrathecal release of inflammatory cytokines, leading to synthesis and release of MMP-9, potentially culminating in insult to brain parenchyma, resulting in release of neuronal and astrocytic degradation products and culminating in MRI verifiable lesions and clinical states of brain deficiency. Interestingly, a recent study [42] indicated that IgG dose-dependently downregulates secretion of MMP-9 by macrophages, suggesting a possible early therapeutic manipulation in NPSLE.

## Competing interests

None declared.

## Abbreviations

CNS = central nervous system; CSF = cerebrospinal fluid; IL = interleukin; MMP = matrix metalloproteinase; MRI = magnetic resonance imaging; NPSLE = neuropsychiatric involvement in systemic lupus erythematosus; SLE = systemic lupus erythematosus.

## Figures and Tables

**Table 1:** Clinical central nervous system manifestations in systemic lupus erythematosus patients included in the study

CNS manifestations	NPSLE (*n *= 43)	No NPSLE (*n *= 71)	aPL syndrome (*n *= 9)
Acute confusional state	1	1	0
Anxiety disorder	0	5	0
Aseptic meningitis	1	0	0
Cerebrovascular disease	6	8	4
Cognitive dysfunction	9	19	1
Demyelinating syndrome	4	1	0
Headache	8	21	4
Mood disorders	5	11	1
Movement disorder	2	0	3
Myastenia gravis	0	2	0
Myelopathy	1	1	0
Polyneuropathy	0	1	0
Mononeuropathy	0	1	0
Neuropathy	0	1	0
Psychosis	7	1	0
Seizure disorders	6	2	0
Tiredness	0	8	0

Each patient may have had multiple clinical manifestations of central nervous system (CNS) involvement. aPL, antiphospholipid; NPSLE, neuropsychiatric involvement in systemic lupus erythematosus.

**Table 2:** Pharmacological treatment of patients with systemic lupus erythematosus included in the study at the time the lumbar puncture was performed

Treatment	NPSLE (*n *= 43)	No NPSLE (*n *= 71)	aPL syndrome (*n *= 9)
Prednisolone (≤10 mg)	18	37	3
Prednisolone (>10 mg)	7	8	0
No prednisolone	18	23	5
Antimalarials	1	8	0
Azathioprine	5	10	3
Azathioprine + cyclosporin A	2	3	0
Azathioprine + antimalarials	0	0	0
Methotrexate	5	1	0
Cyclosporin A	0	6	1
Cyclophosphamide	14	10	0
Cyclophosphamide + cyclosporin A	3	0	0
Cyclophosphamide + antimalarials	0	1	0
No cytotoxic drug	11	30	4
Antihypertensive treatment	14	15	2
Low-dose aspirin	8	19	3
Warfarin	3	2	2

aPL, antiphospholipid; NPSLE, neuropsychiatric involvement in systemic lupus erythematosus.

**Table 3:** Relationship between intrathecal expression of matrix metalloproteinase-9 and inflammatory cytokines, and astrocytic and neuronal degradation products in 119 patients with systemic lupus erythematosus

	MMP-9	
	
	r	*P*
MMP-2	0.40	<0.0001
IL-6	0.30	0.002
IL-8	0.47	<0.0001
Tau	0.26	0.007
GFAP	0.33	<0.04

GFAP, glial fibrillary acidic protein; MMP, matrix metalloproteinase.
